# Recurrent Enterolithiasis Small Bowel Obstruction: A Case Seldom Described

**DOI:** 10.1155/2017/4684182

**Published:** 2017-05-14

**Authors:** Ashish Lal Shrestha, Pradita Shrestha

**Affiliations:** Department of General Surgery, United Mission Hospital, Tansen, Palpa, Nepal

## Abstract

**Background:**

Enterolithiasis of the small bowel is a rare phenomenon in humans although it has been frequently described in equines. Primary enteroliths have been described including those occurring secondary to conditions like Crohn's disease, small bowel diverticula, tuberculous or postoperative strictures, and blind loops but those occurring in an otherwise normal gut are exceedingly rare. Of even greater rarity is a recurrent small bowel enterolith presenting with obstruction. This may be the first report of such kind.

**Case Presentation:**

A 70-year-old man undergoing treatment for stable alcoholic liver disease presented to the emergency with gradually progressive diffuse abdominal pain associated with vomiting and constipation for 7 days. He had gaseous abdominal distention but was not obstipated. He had a history of 2 laparotomies in the past for small bowel obstruction secondary to enterolith impaction. He was initially managed conservatively but since there was no significant clinical improvement, he underwent an exploratory laparotomy. A recurrent enterolith 5 × 5 cm in size was found impacted in the mid ileum with multiple dense serosal adhesions and bands. Adhesiolysis and enterotomy with removal of enterolith were performed.

**Conclusion:**

Recurrent enterolithiasis of the small bowel is a rare phenomenon and may present with recurrent obstruction. Definitive preoperative diagnosis is not always possible and a high index of suspicion is required to avoid table misdiagnosis. Surgery is the mainstay of treatment once conservative measures fail. Laparoscopic methods may help in diagnosis and avoid possibility of a subsequent adhesive bowel obstruction but are associated with technical challenges.

## 1. Introduction

The term “enterolith” is applied to the calculi that form within the intestinal lumen. Though common in equine population, these are cases of rarity in humans. The first reported case was that by Chomelin J in 1710 in Historie de l'Academie Royal in an autopsy report with a duodenal diverticulum [1]. Pfahler and Stamm are credited for the first radiologic description of alimentary stone in 1915 [1].

Gut hypomotility or stasis is thought to be the causal factor for this, the usual contents being inspissated fecal matter, calcium phosphates, magnesium, bacteria, epithelial debris, and unconjugated choleic acid with little or no cholesterol [1–4].

We report an interesting case of a rare recurrent small bowel enterolithiasis presenting with recurrent obstruction. Its clinical presentations, investigative findings, and management are discussed and relevant literatures are reviewed. The rarity of this case is the unusual mode of presentation of an unusual disease.

## 2. Case Presentation

A 70-year-old man previously being treated for stable alcoholic liver disease presented with gradually progressive diffuse abdominal pain associated with vomiting and constipation for 7 days. Physical examination revealed gaseous abdominal distention without tenderness or mass. He had a history of 2 laparotomies in the past both for small bowel obstruction secondary to enterolith impaction that had failed to resolve with conservative measures.

The finding on first operation 3 years ago was that of a 3 × 5 cm obstructing enterolith in the ileum 20 cm proximal to the ileocaecal junction. This was removed through an enterotomy and the affected segment of ileum was resected with primary end to end anastomosis. There were no diverticula or any other inciting factors identified. The histopathology of the resected small bowel was reported to have no specific findings.

Following this, he presented 2 years later with similar symptoms. On second operation the findings were again similar to that of the first operation with an impacted enterolith in the mid ileum along with minimal adhesions. He underwent enterotomy and removal of the enterolith. Following the second operation he was asymptomatic till this presentation.

At the current presentation, his hematological and biochemical workup was normal and abdominal radiographs were inconclusive. USG revealed a normal study.

He was initially managed conservatively in lines of adhesive bowel obstruction.

In view of patient's general condition and lack of facilities, CT scan and endoscopy could not be done.

**Figure 1:**
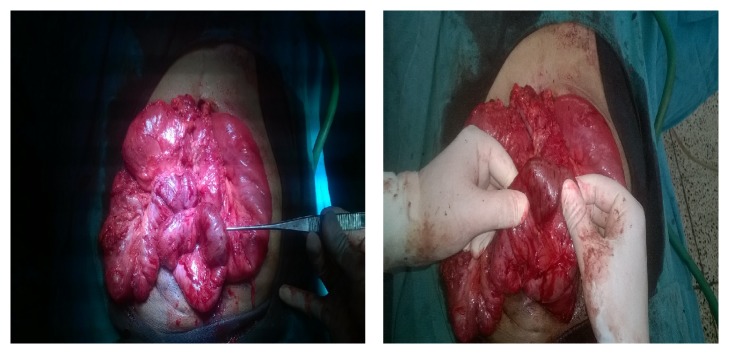
Intraoperative appearance of enterolith impacted in the mid ileum prior to enterotomy with multiple dense adhesions and bands.

**Figure 2:**
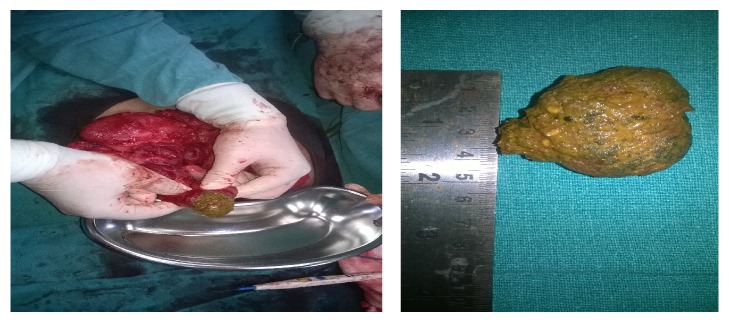
Intraoperative appearance of enterolith impacted in the mid ileum following enterotomy.

After a mild initial symptomatic improvement, he developed gradual and progressive abdominal distention with pain and obstipation. Suspecting adhesive obstruction and keeping in mind the possibility of a recurrent enterolith bowel obstruction, he was taken for an exploratory laparotomy. On table findings were those of a recurrent enterolith 5 × 5 cm in size impacted in the mid ileum with multiple dense serosal adhesions and bands as shown in Figures 1 and 2. Apart from this no other abnormal findings were identified.

The enterolith was disimpacted through an ileal enterotomy followed by primary closure of the enterotomy.

The enterolith was not sent for biochemical analysis considering that it may not contribute to additional information from management point of view.

His subsequent postoperative course was stormy and developed burst abdomen on 8th postoperative day that required mass closure. But following this he showed gradual and steady improvement. After a total stay of 6 weeks, he was discharged in a stable state and had improved on follow-up visit at 3 months. At follow-up he was advised to avoid high roughage diet and consume stool softeners on PRN basis thinking that this would help him avoid another similar episode.

## 3. Discussion

In 1947, Grettve classified enteroliths into primary and secondary types based on their source: primary-inside the bowel and secondary-outside the bowel (associated organs like gall bladder) [1, 5].

Primary enteroliths can occur secondary to conditions like Crohn's disease, small bowel diverticula, tuberculous or postoperative strictures, and blind loops [2, 6–8]. But enterolith occurring in an otherwise normal gut is rarely described and even rarer is recurrent enterolith causing small bowel obstruction [4]. In fact, this may be the first report of a recurrent enterolith. An extensive search in PubMed, Medline, and Google in reference to recurrent enterolithiasis did not show any case reports from 1950 till now.

In 1960, Atwell and Pollock further classified primary enteroliths into true and false based on the chemical analysis of stone composition and their location [1, 2].

The true types are uncommon and formed by the precipitation of alimentary chime while the false types result from the agglutination of indigestible foreign materials like seeds, bone, vegetable material (phytobezoar), hair (trichobezoar), and so forth [2].

Specific preoperative radiological diagnosis of enteroliths is difficult even with water soluble contrast study combined with a cross sectional imaging like CT [4]. Plain radiographs may suggest the probable level of obstruction but may not reveal specific shadows, making it a diagnostic surprise on table. Probably owing to low calcium content in these stones, they are rarely picked up in plain X-rays [9, 10]; on the other hand findings like pneumobilia on the same could suggest gallstone ileus that must be supported with findings of biliary abnormalities on ultrasonography [11].

Definitive treatment is surgical [1]. The consensus management policy at laparotomy is to first attempt manual lysis of the calculus without enterotomy and then milking the smaller parts into proximal colon allowing exit via rectum [1, 12]. If this is not possible, enterotomy removal from a proximal less edematous portion can be done [1, 6]. Bowel resection and anastomosis are usually done if there is a coexistent severe inflammation, perforation, necrotic bowel diverticulosis, or long segment or multiple strictures causing enterolithiasis [1, 2].

The options of minimally invasive techniques may be explored especially if preoperative diagnosis can be made, in order to avoid future adhesions if recurrent enteroliths can be kept in mind as a distant possibility.

## 4. Conclusion

In conclusion, recurrent small bowel enterolithiasis with obstruction is an uncommon presentation of a rare diagnosis. The clinical and radiological picture may not always lead to a definitive diagnosis preoperatively and many a time this may be possible only on table. An accurate preoperative diagnosis requires a high index of clinical suspicion. Once conservative measures fail, surgical treatment remains the mainstay of management.

## Abbreviations

CT: Computed tomography
USG: Ultrasonography of the abdomen and pelvis
PRN: Pro re nata (as and when required).
